# Increased Litter Size and Suckling Intensity Stimulate mRNA of *RFamide-related Peptide* in Rats

**DOI:** 10.22074/ijfs.2015.4554

**Published:** 2015-10-31

**Authors:** Atefeh Noroozi, Mohammad Reza Jafarzadeh Shirazi, Amin Tamadon, Ali Moghadam, Ali Niazi

**Affiliations:** 1 Department of Animal Sciences, College of Agriculture, Shiraz University, Shiraz, Iran; 2 Transgenic Technology Research Center, Shiraz University of Medical Sciences, Shiraz, Iran; 3 Biotechnology Institute, College of Agriculture, Shiraz University, Shiraz, Iran

**Keywords:** *RFRP* mRNA, Suckling Intensity, Dorsomedial Hypothalamic Nucleus, Lac- tation, Rat

## Abstract

**Background:**

RFamide-related peptide-3 (RFRP-3) inhibits gonadotropin releasing hormone (GnRH) and luteinizing hormone (LH) secretion in rats. This study evaluates the effects of litter size and suckling intensity on *RFRP* mRNA expression in the dorsomedial hypothalamic nucleus (DMH) of rats.

**Materials and Methods:**

A total of 32 pregnant and 4 non-lactating ovariectomized (control group) Sprague-Dawley rats were used in this experimental study. Lactating rats were allotted to 8 equal groups. In 3 groups, the litter size was adjusted to 5, 10, or 15 pups upon parturition. Dams were allowed to suckle their pups continuously until 8 days postpartum. In the other 3 groups, the litter size was adjusted to 5 pups following birth. These pups were separated from the dams for 6 hours on day 8 postpartum, after which the pups were allowed to suckle for 2.5, 5, or 7.5 minutes prior to killing the dams. In 2 groups, lactating rats with 10 and 15 pups were separated from their pups for 6 hours on day 8 postpartum. In these groups, the pups were allowed to suckle their dams for 5 minutes before the dams were killed. All rats were killed on day 8 postpartum and the DMH was removed from each rat. We evaluated *RFRP* mRNA expression using realtime polymerase chain reaction (PCR).

**Results:**

The expression of *RFRP* mRNA in the DMH increased with increased litter size and suckling intensity compared to the controls. The effect of suckling intensity on the expression of *RFRP* mRNA was more pronounced compared to the litter size.

**Conclusion:**

Increased litter size and suckling intensity stimulated *RFRP* mRNA expression in the DMH which might contribute to lactation anestrus in rats.

## Introduction

Gonadotropin releasing hormone (GnRH) is a hypothalamic neuropeptide that acts as the primary signal for regulation of gonadotropins, luteinizing hormone (LH) and follicle-stimulating hormone (FSH) secretion. It is well established that GnRH acts as a key neurohormone for vertebrate reproduction. 

Gonadotropin-inhibitory hormone (GnIH) is a key inhibitory regulator of the hypothalamus-pituitary-gonads axis. This hormone has been shown to directly act on the pituitary gland and inhibit gonadotropin release (1). Initially identified in quails, this was the first demonstration that a hypothalamic neuropeptide could inhibit gonadotropin release in any vertebrate. GnIH has since been isolated as a mature peptide in starlings (2) and zebra finches (3). 

RFamide-related peptides (RFRP) are GnIH orthologs that have been subsequently identified in a number of other vertebrates, including mammals. In mammals, cDNAs that encode LPXRFamide peptides (X=L or Q) similar to GnIH were investigated by a gene database search (4). The cDNAs identified from the mammalian brain encode RFRP-1, 2, and 3, in cattle and humans, as well as RFRP-1 and 3 in rodents (5,7). RFRP-3 has been shown to putatively modulate the negative feedback effect of estrogen on gonadotropin secretion (8). RFRP-ir cells cluster in the dorsomedial hypothalamic nucleus (DMH) and have been identified in hamsters, rats, and mice (8). The inhibitory effects of RFRP-3 on gonadotropin release were reported in rodents (9,10) and sheep (11,12). 

Follicular maturation and ovulation are inhibited during lactation in various mammals (13). Inhibition of the estrous cycle in lactating rats mostly results from inhibition of LH and GnRH secretion (14). Although suckling is an important inhibitory cue for LH surge during the first 8 days of lactation in rats, separating pups from their dams restores LH secretion (15). Levels of LH pulsatile secretion are low in lactating rats (16) and humans (17). Administration of bromocriptine (a dopamine agonist used in the treatment of hyperprolactinemia) does not impact the inhibitory effect of suckling on LH pulse (18). Research has shown that endogenous opioid peptides do not mediate suppression of the LH release by the suckling stimulus. In the rat model, intravenous injection of naloxone does not increase LH secretion during early lactation (19). Neuroendocrine mechanisms that affect inhibition of LH secretion during lactation are unknown (20). 

Suckling is an appropriate model for studying the reproductive endocrine hormones involved during lactation and to investigate the neuroendocrine pathways that regulate negative energy balance. Estrus and ovulation are delayed for approximately 20 days in lactating rats that suckle 6 to 10 pups. The delay period is dependent on the number of pups. If the number of pups is greater than 12 with a 2-day-long water and food withdrawal period, then the strength of suckling is increased (21). Therefore, the number of pups and strength of suckling may be critical stimulants in inhibiting LH surge. The aims of the present study are to evaluate the effects of litter size and suckling intensity on *RFRP* mRNA transcription in lactating rats. The findings will be beneficial to better clarify the underlying mechanism(s) involved in lower reproductive performance attributed to lactation anestrus. 

## Materials and Methods

### Animals, experimental groups, and sampling

In the present experimental study, we randomly selected 32 pregnant and 4 ovariectomized (3-4 month-old) female Sprague-Dawley rats (* Rattus norvegicus*) that weighed 205.9 ± 10.7 g (mean ± SD). Rats were housed in individual cages under controlled temperature (22 ± 2˚C) and light (14 hours light/10 hours dark; lights on from 07:00 to 21:00) with free access to food and water in the Laboratory Animal Center of Shiraz University of Medical Sciences, Shiraz, Iran. The rats were treated humanely and in compliance with the recommendations of the Animal Care Committee at Shiraz University of Medical Sciences. The rats were randomly assigned to 9 groups (n=4 per group). The control group comprised 4 ovariectomized rats. Each rat assigned to the ovariectomized group received an intraperitoneal injection of ketamine (100 mg/kg, Netherlands) and xylazine (7 mg/kg, Alfazyne, Netherlands) as anesthesia. Control rats were ovariectomized through the ventral midline incision. Further procedures were carried out over a two-week recovery period. 

Lactating rats were allotted to 8 groups (n=4 per group). These rats were allowed to suckle their pups until day 8 postpartum. In 3 groups, the litter sizes were adjusted to 5, 10, or 15 pups upon parturition. Rats from these groups were allowed to suckle their pups continuously. In an additional 3 groups of rats, the litter size was adjusted to 5 upon birth. The pups were separated from their dams on day 8 postpartum for 6 hours, after which they were allowed to suckle their dams for 2.5, 5, or 7.5 minutes before the dams were killed. This separation time was selected according to Marina et al. (22). This time period made the pups hungry which enabled them to intensively suckle their mothers’ teats. The minimum of 2.5 minutes and 2.5-minute intervals were selected according to the minimum time in increase in RNA levels immediately detected after transcription stimulation of a single cell (23). Two groups of lactating rats with either 10 or 15 pups were similarly separated from their pups for 6 hours on day 8 postpartum, after which the pups were allowed to suckle their dams for 5 minutes before the dams were killed. Rats were anesthetized with ether and killed via cervical dislocation at 15:00 to 16:00 on day 8 postpartum. Brains were immediately removed and the diencephalon was dissected out by an anterior coronal section, anterior to the optic chiasm, and a posterior coronal cut at the posterior border of the mammillary bodies. To separate DMH, a third coronal cut was made through the middle of the optic tract, just rostral to the infundibulum (24). The specimens were stored in liquid nitrogen until further analysis. 

### Real-time polymerase chain reaction (PCR)

Total RNA was extracted using RNX-Plus buffer (Cinnagen, Tehran, Iran). Briefly, the tissue (100 mg) was ground in liquid nitrogen, transferred to RNX-Plus buffer (1 mL) in an RNase-free microtube, mixed thoroughly, and kept at room temperature for 5 minutes. Chloroform (0.2 mL) was added to the slurry and mixed gently. The mixture was centrifuged at 12000×g (4˚C) for 20 minutes after which the supernatant was transferred to another tube, then precipitated with an equal volume of isopropanol for 15 minutes. The RNA pellet was washed with 75% ethanol, quickly dried, and re-suspended in 50 μL RNase-free water. The integrity and quantity of RNA was checked by visual observation of 28S and 18S rRNA bands on a 1.2% agarose gel. The purified total RNA was quantified by a Nano-Drop ND 1000 spectrophotometer (Nano-Drop Technologies, Wilmington, DE, USA). DNase treatment was carried out using a DNase kit (Fermentas, St. Leon-Roth, Germany) according to the manufacturer’s instructions. The DNase-treated RNA (3 μg) was used for first strand cDNA synthesis with 100 pmol oligo-dT, 15 pmol dNTPs, 20 U RNase inhibitor, and 200 U MMulv reverse transcriptase (Fermentas, Germany) in a final volume of 20 μL. Primers were designed using Allele ID 7 software (Premier Biosoft International, Palo Alto, USA) for the reference gene and RFRP (NM_023952). The rat *Glyceraldehyde- 3-phosphate dehydrogenase (GAPDH)* gene (M32599) was used as a reference gene for data normalization (Table 1). Relative real-time PCR was performed in a 20 μL volume that contained 1 μL cDNA, 1X Sybr Green buffer and 4 pmol of primer. The amplification reactions were carried out in a Line-Gene K thermal cycler (Bioer Technology Co., Ltd., Hangzhou, China) under the following conditions: 2 minutes at 94˚C, 40 cycles of 94˚C (10 seconds), 57˚C (15 seconds), and 72˚C (30 seconds). After 40 cycles, the specificity of the amplifications was tested by heating from 50˚C to 95˚C, which resulted in melting curves. All amplification reactions were repeated three times under identical conditions, including a negative control and five standard samples. To ensure that the PCR products were generated from cDNA rather than genomic DNA, proper control reactions were implemented in the absence of reverse transcriptase. For quantitative real-time PCR data, the relative expression of *RFRP* mRNA was calculated based on the threshold cycle (C_T_) method. The C_T_ for each sample was calculated, using Line-gene K software (25). Accordingly, the fold expression of the target mRNAs over the reference values was calculated by the equation 2^-ΔΔCT^ (26), where ΔC_T_ was determined by subtracting the corresponding GAPDH C_T_ value (internal control) from the specific C_T_ of the target (*RFRP*). The ΔΔC_T_ was obtained by subtracting the ΔC_T_ of each experimental sample from that of the calibrator one (non-lactating ovariectomized rats).

**Table 1:** Real-time polymerase chain reaction (PCR) primer sequences used to evaluate relative expression of *RFRP* gene in a rat model



*RFRP-F*	5΄ CTCAGCAGCCAACCTTCC 3΄	165
*RFRP-R*	5΄AAACCAGCCAGTGTCTTG3΄	
*GAPDH-F*	5΄AAGAAGGTGGTGAAGCAGGCATC 3΄	112
Primer	Sequence	Ampliconlength(bp)
*GAPDH-R*	5΄CGAAGGTGGAAGAGTGGGAGTTG3΄	


*GAPDH; Glyceraldehyde-3-phosphate dehydrogenase* and *RFRP; RFamide-related peptide-3*.

### Statistical analysis

Data from relative expression of the *RFRP* gene were subjected to the test of normality and analyzed by one-way ANOVA (SPSS for Windows, version 11.5, SPSS Inc., USA). Mean separation was performed by post hoc LSD test at P=0.05.

## Results

**Fig.1:**
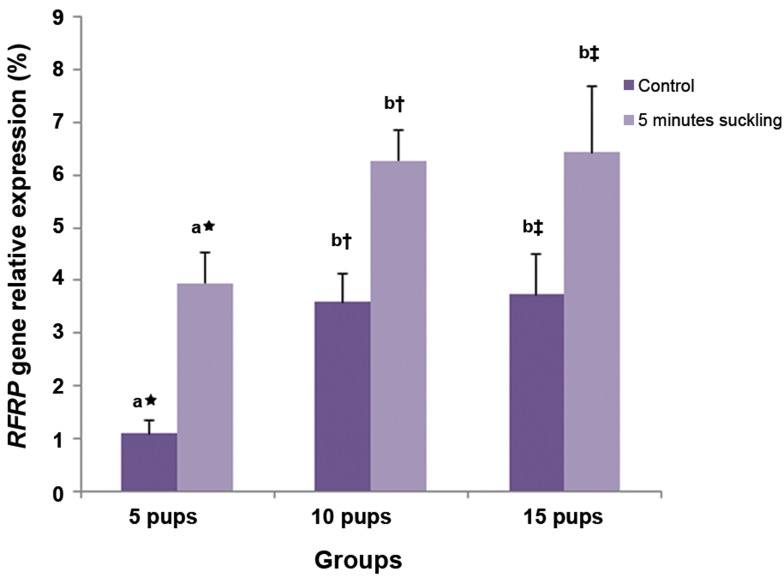
The effect of litter size and 5 minutes suckling duration (after a 6-hour separation period of the dam and pup) on relative expression of the *RFamide-related peptide-3 (RFRP)* gene (mean ± SE) in the dorsomedial hypothalamic nucleus (DMH) of lactating rats (n = 4) with 5, 10, or 15 pups. Control lactating rats were not separated from their pups. Different letters indicate significant differences between different litter sizes in each group and the same symbols indicate significant differences between the different suckling durations with the same litter size (P<0.05).

**Fig.2:**
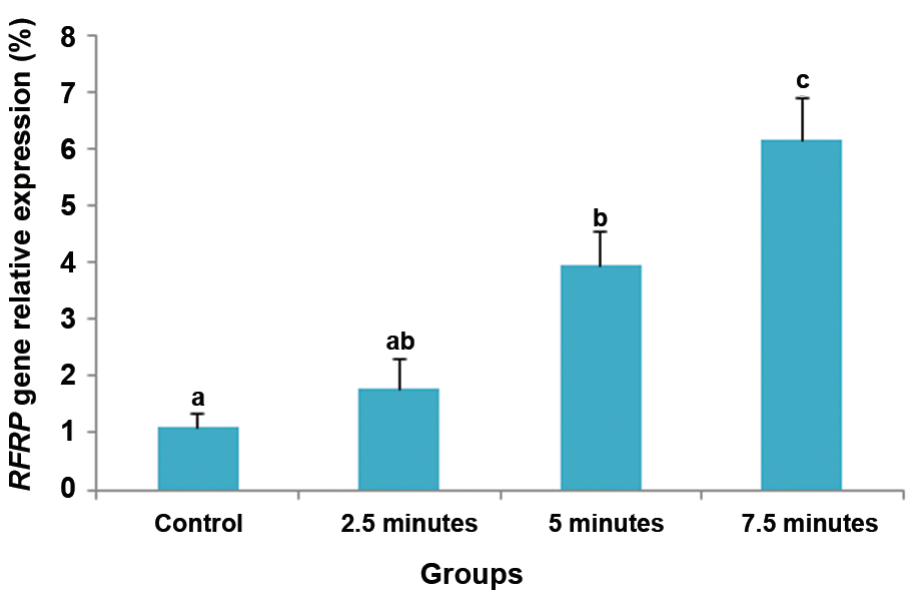
The effect of suckling intensity on relative expression of the *RFamide-related peptide-3 (RFRP)* gene (mean ± SE) in the dorsomedial hypothalamic nucleus of lactating rats (n=4) with 5 pups which were separated from their pups for 6 hours on day 8 postpartum, after which the pups were allowed to suckle their dams for 2.5, 5, or 7.5 minutes. Control lactating rats were not separated from their pups. Different letters indicate significant difference (P<0.05).

The relative expression of *RFRP* mRNA in rats continuously housed with five pups was lower than those that suckled either 10 or 15 pups (P=0.02, Fig.1). The relative expressions of *RFRP* mRNA in the DMH of lactating rats continuously housed with 5 (P=0.009), 10 (P=0.01), or 15 (P=0.02) pups were respectively lower than in rats that were separated from their pups for 6 hours, then allowed to suckle for 5 minutes (Fig.1). The relative expression of *RFRP* mRNA in lactating rats with 5 pups that were separated from their pups, then allowed to suckle for 5 minutes was lower than those that suckled either 10 or 15 pups (P=0.02, Fig.1). The relative expression of *RFRP* mRNA in the DMH of lactating rats with 5 pups that were separated from their pups for 6 hours, then allowed to suckle for 2.5 (P=0.001) or 5 (P=0.03) minutes was lower than in rats that suckled for 7.5 minutes (Fig.2).

## Discussion

In this study, *RFRP* mRNA levels greatly increased in the DMH during suckling. The *RFRP* mRNA in neurons of DMH from lactating rats increased with increased numbers of suckling pups and intensity of suckling. Consistent with our findings, *RFRP* mRNA expression (27) and RFRP-3ir neurons According to immunohistochemistry analyses (28) in DMH of the hypothalamus of lactating rats was more than non-lactating rats. It has been shown that the effects of RFRP-3 were opposite to kisspeptin during the estrous cycle in the rat (29). In keeping with our findings, Yamada et al. (30) reported that suckling stimulus inhibited the expression of kisspeptin in neurons of the arcuate nucleus (ARC). These findings demonstrated the inhibitory effect of RFRP-3 on reproduction at transcription and translation levels during lactation in a rat model. 

The present study showed that increased intensity of suckling resulted in higher expression of *RFRP* mRNA in the DMH. RFRP-1 is a secretion stimulator of prolactin in rats (4). A relationship was observed between the intensity of the suckling-induced prolactin increase and litter size in rats (31) and level of increase in prolactin secretion during lactation. Increased prolactin levels directly inhibited GnRH and LH (32) secretions. Prolactin did not mediate the suppressing effect of the suckling stimulus on LH secretion at the hypothalamic level in rats during early (33) and mid-lactation (18). In support of our results, Hinuma et al. (4) reported that intracerebroventricular RFRP-1 administration caused increased prolactin release in humans. It was likely that the negative effects of prolactin on LH were exerted through RFRP-3. 

Tuberoinfundibular dopaminergic (TIDA) neurons in ARC are known as the key regulators of prolactin release (34). Dopamine has been shown to inhibit prolactin. A close contact between RFRP neurons and dopamine was reported where RFRP receptors were expressed in dopamine neurons (35). Therefore, RFRP-3 might stimulate prolactin secretion by suppressing the activity of dopamine neurons. 

Intensive suckling acutely increased *RFRP* mRNA expression, whereas this effect was not observed with continuous suckling. Lactating rats normally receive continuous suckling from pups rather than intensive suckling. The intensity of the suckling stimulus was reported to depend on the number of pups attached to the nipples, duration of attachment and the suckling intensity (36). In the present study, increased duration of attachment (5 and 7.5 minutes) of hungry pups acutely increased *RFRP* mRNA expression after 5 minutes of intensive suckling with the same litter size (5 pups). Increased numbers of hungry pups after 5 minutes of intensive suckling acutely increased *RFRP* mRNA expression. Consistent with our findings, suckling stimulus inhibited the expression of kisspeptin in ARC neurons (30). There was a negative correlation between expression of kisspeptin and RFRP in rat ARC neurons (29). Therefore, increased litter size and/or duration of suckling caused increased suckling intensity that led to increased *RFRP* mRNA expression in ARC neurons in lactating rats. 

We showed that increased suckling stimulus caused more *RFRP* mRNA expression. Suckling has been shown to be an important inhibitor of LH secretion in lactating rats (37). Although, few data reported the relationship between lactation stress and expression of RFRP-3, it has been shown that stress increased RFRP expression in male rats (38). Cortisol levels were higher in lactating female rhesus macaques than in non-lactating females (37). Corticotrophin-releasing hormone was not critical in conveying the inhibitory inputs of the suckling stimulus in ovariectomized lactating rats (39), but cortisol treatment suppressed LH release in rats (40) and ewes (41). Consequently, increased glucocorticoid secretion due to increased litter size and/or suckling intensity might inhibit gonadotropin secretion through stimulation of RFRP-3. 

Increased numbers of pups per lactating rat result in higher milk production, therefore the negative energy balance becomes more exacerbated (42). Adequate energy reserves are essential for reactivation of the reproductive axis. During periods of negative energy balance, GnRH release is suppressed (43). Regardless of the level of energy intake the efficiency of energy use substantially increases during lactation in rats. The mechanisms involved in negative energy balance play an important role in the change of energy expenditure (42). Melanin-concentrating hormone (MCH) and orexin, two appetite neuropeptides, have been reported to inhibit LH secretion during lactation (33). On the other hand, RFRP neurons project to MCH and orexin producing cells in the lateral hypothalamic area of sheep (44). Therefore, enhancement of *RFRP* mRNA expression with an increase in lactation is simultaneous with negative energy balance and inhibition of reproduction. 

## Conclusion

We demonstrated a relationship between *RFRP* mRNA expression, increased litter size, and suckling intensity in the DMH of rats. 

Stimulation of RFRP-3 might be a factor in inhibition of LH secretion during lactation, although the mechanisms underlying this inhibition should be further addressed. 
